# The Mediating Role of Organizational Silence in the Relationship Between Change Fatigue and Work Engagement Among Chinese Clinical Nurses

**DOI:** 10.1155/jonm/2155118

**Published:** 2026-08-20

**Authors:** Xiangwen You, Haibo Ma, Rong Yan, Miao Yao, Hongyan Lu

**Affiliations:** ^1^ Master Training Station, General Hospital of Ningxia Medical University, Yinchuan, Ningxia Hui Autonomous Region, China, nxmu.edu.cn; ^2^ School of Nursing, Ningxia Medical University, Yinchuan, Ningxia Hui Autonomous Region, China, nxmu.edu.cn; ^3^ Department of Burns and Plastic Surgery, General Hospital of Ningxia Medical University, Yinchuan, Ningxia Hui Autonomous Region, China, nxmu.edu.cn; ^4^ Department of Nursing, General Hospital of Ningxia Medical University, Yinchuan, Ningxia Hui Autonomous Region, China, nxmu.edu.cn

**Keywords:** change fatigue, clinical nurses, mediating effect, organizational silence, work engagement

## Abstract

**Background:**

Healthcare institutions are undergoing ongoing organizational changes to optimize clinical services and patient care. These changes may induce change fatigue and organizational silence among nursing staff, undermining their work attitudes and professional performance and potentially compromising patient safety. While previous studies have explored the relationship between change fatigue, organizational silence, and work engagement, few have examined the mediating effect of organizational silence in this relationship, especially within the Chinese healthcare context.

**Objective:**

This study aimed to examine the mediating role of organizational silence in the relationship between change fatigue and work engagement among clinical nurses.

**Methods:**

This descriptive cross‐sectional study was conducted online from March to May 2025. A total of 1067 registered nurses were recruited from five tertiary public hospitals in Ningxia, China, using the convenience sampling method. Data were collected using the following instruments: (1) a demographic questionnaire, (2) the Change Fatigue Scale, (3) the Organizational Silence Scale, and (4) the Work Engagement Scale. Data analysis was performed using descriptive statistics, Pearson correlation analysis, and structural equation modeling (SEM).

**Results:**

The average scores for change fatigue, organizational silence, and work engagement were 25.40 ± 9.27, 49.76 ± 19.37, and 44.70 ± 13.72, respectively. Change fatigue was positively associated with organizational silence (*r* = 0.440, *p* < 0.001) and negatively correlated with work engagement (*r* = −0.332, *p* < 0.001). Organizational silence was negatively correlated with nurses’ work engagement (*r* = −0.402, *p* < 0.01). The mediation analysis showed that organizational silence played a partial mediating role in the relationship between change fatigue and work engagement, with a mediation effect value of −0.153 (95% CI [−0.087, −0.055]), accounting for 44.1% of the total effect.

**Conclusions:**

This study confirmed significant correlations among change fatigue, organizational silence, and work engagement among Chinese clinical nurses. Moreover, organizational silence exerted a partial mediating effect on the association between change fatigue and work engagement. Nevertheless, causal inferences should be drawn with caution, as this research adopted a cross‐sectional design.

**Implications for Nursing Management:**

To maintain nurses’ work engagement amid organizational changes, nursing managers should implement integrated intervention strategies to alleviate change fatigue and dismantle organizational silence. Such managerial measures help foster a supportive, high‐efficiency environment for healthcare transformation, thereby promoting nurses’ occupational wellbeing and underpinning the long‐term sustainable development of nursing teams.

**Patient or Public Contribution:**

No patient or public contribution.

## 1. Introduction

In the modern healthcare system, organizational changes are primarily driven by the need to enhance health services and meet patients’ evolving expectations [1]. As key implementers of these changes, clinical nurses are an essential component of the healthcare workforce [2]. However, frequent and continuous changes have posed challenges to clinical nursing practice, including increased workload, persistent uncertainty, role ambiguity, and staffing shortages [3–5]. Such adverse working environments precipitate occupational burnout, poor professional wellbeing, and increased nurse turnover intent [6]. Hence, against the background of rapid healthcare system changes, identifying predictive antecedents of nurses’ work engagement is critical for nursing administration.

Work engagement is a critical indicator of nurses’ work attitudes and efficiency, which is defined as an active, positive, and fulfilling work‐related psychological state characterized by three core dimensions: vigor, dedication, and absorption [7, 8]. As a focus of positive psychology research, it has been widely introduced into the nursing management [9]. Prior studies have shown that nurses with higher work engagement exhibit greater attentiveness and patience toward patients, achieve better job performance, and report lower turnover intentions [10–12]. Conversely, insufficient work engagement among nursing staff undermines patient care, elevates team stress, and compromises the operational efficiency of healthcare services. Previous studies have identified work overload, illegitimate tasks, psychosocial safety climate, and transformational leadership as key factors influencing nurses’ work engagement [6, 11, 13, 14]. Despite explorations of the factors influencing nurses’ work engagement from various perspectives, investigating its antecedents remains an important topic, particularly through the lens of organizational change [15].

It is worth noting that healthcare systems are constantly evolving, making change fatigue and organizational silence pervasive issues among nursing staff. While previous studies have identified close correlations among change fatigue, organizational silence, and work engagement [16, 17], empirical research that integrates all three variables in nurse populations remains insufficient. To fill this research gap, the Job Demands‐Resources (JD‐R) model provides a robust framework for understanding nurses’ work engagement [18]. It posits that all job characteristics can be divided into two core categories: job demands and job resources. Job demands are defined as work‐related aspects that require sustained physical or psychological effort. Examples include frequent and continuous change, escalated workload, and sense of uncertainty amid change. Job resources refer to factors that mitigate the adverse effects of job demands, facilitate the achievement of work goals, and foster personal growth. Examples include organizational support, open communication channels, and opportunities for participatory decision‐making.

Change fatigue refers to a state of mental and emotional exhaustion, diminished personal initiative, and passive acquiescence to organizational changes, resulting from the cumulative stress of frequent, poorly managed, or disjointed change initiatives [19]. In the nursing profession, change fatigue has been widely discussed and is increasingly recognized as a critical barrier to the successful implementation of organizational change. Research has shown that frequent organizational restructuring and policy shifts are strongly associated with elevated levels of change fatigue among nursing staff [20]. Moreover, nurses experience change fatigue at a rate of approximately three times higher than other healthcare professionals [21]. Within China’s healthcare system, frontline nurses face relentless healthcare reforms and adjustments, making change fatigue a prevalent workplace challenge among nursing staff [22]. This issue has attracted attention due to its chronic, erosive impacts on nurses’ professional well‐being and the sustainable development of nursing teams [23]. According to the JD‐R model, work engagement emerges from the dynamic interplay between these two categories of job characteristics. Frequent organizational changes constitute a major job demand, compelling nurses to invest substantial personal resources to adapt. In an imbalance of high demands and low resources, such fatigue not only hinders an individual’s ability to achieve work goals but also exacerbates physical and psychological depletion. In an effort to safeguard their professional roles, employees may opt to reduce their work engagement, consequently prompting counterproductive behaviors such as negativity and disengagement. Therefore, based on the above analysis, we propose the following hypothesis:

H1: There is a significant negative relationship between change fatigue and work engagement among clinical nurses.

Organizational silence refers to the phenomenon where nurses intentionally withhold work‐related insights, concerns, or constructive suggestions when confronted with organizational issues or challenges [24]. The dimensions of organizational silence are complex, typically classified based on underlying motivations, including acquiescent silence, defensive silence, and prosocial silence [25]. The successful implementation of organizational change inherently relies on staff input, genuine buy‐in, and active voices from nursing professionals [26]. However, research indicates that nearly 91% of the nurses have reported experiencing organizational silence in their professional practice [27]. Such silence among nurses may manifest as reluctance to report change‐related errors, propose targeted improvement strategies, or passively comply with top‐down directives without voicing dissent [28]. In China, this propensity toward silence is further reinforced by deeply rooted Confucian values of deference to authority and hierarchical workplace norms [29]. This silence behavior impedes information sharing and critical feedback exchange among nurses, nurse managers, and interprofessional colleagues. Furthermore, the inability to detect and correct errors in a timely fashion creates suboptimal decisions and ultimately undermines the implementation of organizational change initiatives across healthcare organizations [30]. Zhu et al. found that higher levels of organizational silence among nurses are significantly associated with lower levels of work engagement [17]. Nurses rely heavily on their colleagues for support, information sharing, and collaborative patient care. When organizational silence occurs, it impedes their communication, leading to poor interpersonal relationships and reduced access to job resources. This in turn affects their autonomy, problem‐solving ability, and task completion efficiency, ultimately decreasing work engagement and indirectly impacting patient care and safety. Based on this rationale, we propose the following hypothesis:

H2: There is a significant negative relationship between organizational silence and work engagement among clinical nurses.

This study also draws on the assumptions of the Conservation of Resources (COR) theory as a supplement to the JD‐R model. This theory provides another theoretical lens to understand why nurses reduce their work engagement in response to workplace stressors such as change fatigue and silence. The COR theory posits that individuals are inherently motivated to obtain, retain, nurture, and protect valued personal and work‐related resources in response to both potential and actual threats of resource loss [31]. It suggests that when nurses experience resource depletion due to the continuous strain of frequent organizational changes, they may strategically adopt organizational silence as a protective coping mechanism to conserve their physical and psychological resources [32, 33]. Therefore, we propose the following hypothesis:

H3: There is a significant positive relationship between change fatigue and organizational silence among clinical nurses.

Organizational silence may function as a key behavioral mechanism linking change fatigue to clinical nurses’ work engagement. Although short‐term silence helps nurses preserve limited personal resources as a passive coping choice, prolonged silence paradoxically depletes their psychological resources [34]. It impedes open communication and cuts off access to restorative positive feedback and social support, triggering a vicious cycle of continuous resource loss that prevents nurses from maintaining stable high work engagement. Taken together, change fatigue operates as a major psychological stressor, and its co‐occurrence with organizational silence contributes to declining work engagement. Hence, we hypothesize the following:

H4: Organizational silence mediates the relationship between change fatigue and work engagement.

Previous studies have separately validated the correlations of change fatigue and organizational silence with work engagement in nurses, yet the internal mechanisms have rarely been explored in China’s nursing setting, especially the mediating role of organizational silence between change fatigue and work engagement. Therefore, the purpose of this study was to explore the mediating role of organizational silence in the relationship between nurses’ change fatigue and work engagement. The findings are expected to provide theoretical support for alleviating nurses’ change fatigue, eroding the culture of organizational silence, boosting the efficiency of healthcare changes, and ultimately safeguarding patient safety.

## 2. Methods

### 2.1. Design

A descriptive cross‐sectional research design was conducted in this study. The study was conducted and reported in accordance with the Strengthening the Reporting of Observational Studies in Epidemiology (STROBE) guidelines.

### 2.2. Participants and Sample

A convenience sampling method was employed to recruit participants from five Grade A tertiary public hospitals in Ningxia, China. The inclusion criteria of participants were as follows: (a) registered nurses who worked in clinical frontline, (b) full‐time nurses with at least 1 year of professional experience in their current work unit, and (c) agreeing to participate in the study. Exclusion criteria included (a) nurse interns and (b) nurses on leave.

This study calculated the sample size based on the likelihood ratio test (LRT) formula developed by Jobst et al. [35], which is widely used for structural equation modeling (SEM), expressed as *T* = (*N* − 1) ∗ *F*. For a‐priori power calculation, we explicitly assumed a population RMSEA of 0.05 as the effect size, with an alpha level of 0.05, df = 18, and a desired statistical power of 80%, the minimum required sample size was determined to be 450. Considering a 10% rate of invalid responses, the final planned sample size was determined to be 500 participants. A total of 1185 questionnaires were collected, of which 1067 were valid (78 questionnaires completed in less than 180 s and 40 questionnaires were straight‐lining responses), with an effective response rate of 90.04%. Furthermore, post hoc power analysis for the hypothesized indirect mediating effect was conducted using the semPower package in R software. Based on the final valid sample of 1067, the achieved statistical power for the indirect effect was calculated to be 0.99, which exceeded the threshold of 0.8, demonstrating that the sample size was adequate.

### 2.3. Study Instruments

#### 2.3.1. Demographic Questionnaire

This questionnaire was self‐developed by the researchers with reference to relevant literature, collecting six variables: gender, age, educational status, marital status, professional title, and years of working.

#### 2.3.2. Change Fatigue Scale

The scale was originally developed by American human resource management experts Bernerth et al. [36] and was culturally adapted for Chinese nursing populations by Zhang et al. [37]. This unidimensional scale comprises six items rated on a 7‐point Likert scale (1 = “*strongly disagree*” to 7 = “*strongly agree*”), yielding a total score ranging from 6 to 42. Higher score indicates stronger sense of change fatigue. A sample item is “The organization has introduced too many change measures.” The scale has demonstrated good reliability with Cronbach’s *α* coefficients of 0.850 in non‐nurse populations and 0.918 in Chinese version [37]. In the present study, Cronbach’s *α* coefficient for the overall scale was 0.920.

#### 2.3.3. Organizational Silence Scale

The scale was developed by Chinese scholar Yang [38]. The scale consists of 20 items and 4 subdimensions: acquiescent silence (6 items), defensive silence (6 items), prosocial silence (4 items), and indifference silence (4 items). Each item is rated on a 5‐point Likert scale (1 = “*never*” to 5 = “*always*”). The overall score ranges from 20 to 100, and a higher score indicates a more severe level of organizational silence. A sample item is “Being in a low position with little influence, my opinions or suggestions will not be taken seriously.” In this study, the KMO coefficient was 0.938, which is considered acceptable, and Bartlett’s test of sphericity was significant (*p* < 0.001), confirming that the data were appropriate for factor extraction. A following confirmatory factor analysis indicated that the factor loadings ranged from 0.828 to 0.892, average variance extracted (AVE) ranged between 0.685 and 0.796, the composite reliability was between 0.915 and 0.956, and the Cronbach’s *α* was 0.965 of the overall scale, thus validating the construct validity and reliability of the instrument.

#### 2.3.4. Work Engagement Scale

Originally developed by Bakker et al. [39], the scale has been validated as a reliable measurement tool in the Chinese context by Fong and Ng [40]. This 9‐item scale consists of three subdimensions: vigor (3 items), dedication (3 items), and absorption (3 items). Each item is rated on a 7‐point Likert scale (0 = “*never*” to 6 = “*daily*”), yielding total scores ranges from 0 to 54 and a higher score indicates a better work engagement state. A sample item is “At work, I feel I am highly capable and qualified for my work.” Fong and Ng reported a Cronbach’s *α* coefficient of 0.88 for the scale. In this study, the overall Cronbach’s *α* coefficient was 0.926, with 0. 737 for vigor, 0.905 for dedication, and 0.788 for absorption.

### 2.4. Data Collection and Quality Control

Data were gathered via an online survey between February and May 2025. This approach is widely adopted in nursing research owing to its efficiency and convenience [41]. The project leader approached the nursing departments of five hospitals to explain the study’s purpose and obtain departmental approval for conducting the study. Wenjuanxing (https://www.wjx.cn/), a secure and widely used online survey platform in China that complies with national data privacy regulations, was utilized for data collection. With the support of the nursing managers, eligible nurses received online questionnaire invitations (QR code or electronic link) via WeChat app. Each IP address was restricted to one submission to avoid duplicate responses. All raw data downloaded from Wenjuanxing were encrypted and stored securely by the research team, with access limited exclusively to research members. To ensure data validity, questionnaires were excluded if they met any of the following criteria: (a) survey completion time less than 180 s (as determined through pilot testing) and (b) same answers or same pattern of answers for all items (e.g., repeated answers 1, 2, and 3).

### 2.5. Ethical Considerations

Ethical approval was obtained from the General Hospital of Ningxia Medical University Ethics Committee (Approval No.: KYLL‐2025‐1371). Before gathering data, all participants reviewed and signed an informed consent form detailing the study’s purpose and methodology. Participants were informed that all collected data would be used solely for academic research, kept strictly confidential, and never disclosed or used for commercial purposes. Participants completed the questionnaire anonymously, with no personal identifiable information (e.g., name, employee ID, and phone number) collected throughout the survey. Wenjuanxing adopts encrypted data transmission to protect respondent privacy during submission. The principles of voluntary participation and the right to withdraw at any time without consequence were underscored.

### 2.6. Data Analysis

Data were analyzed using IBM SPSS Statistics 26.0 and R 4.4.2. Descriptive statistics were presented with frequencies, percentages, mean, and standard deviation (*M* ± SD). The conformity of the data to a normal distribution was assessed based on skewness and kurtosis coefficients with a threshold of ±1, indicating acceptable normality. Harman’s single‐factor method was adopted to assess for common method bias. Pearson’s correlation analysis was applied to examine bivariate relationships among the three core measures. SEM was conducted in the R software using the lavaan package, which served to analyze the pathway correlations between change fatigue, organizational silence, and work engagement. The mediation effect test adopted the bootstrap technique, which calculated the 95% confidence interval (CI) through 5000 repeated samples. The mediation effect was considered statistically significant if the 95% CI did not include zero. The structural model results were illustrated through a path diagram. Model fit was evaluated using multiple goodness‐of‐fit indices (GFIs), including the chi‐square (*χ* ^2^) test, root mean square error of approximation (RMSEA) ≤ 0.08 (or ≤ 0.06 for close fit), standardized root means square residual (SRMR) ≤ 0.08, GFI ≥ 0.90, adjusted GFI (AGFI) ≥ 0.90, comparative fit index (CFI) ≥ 0.90, Tucker–Lewis index (TLI) ≥ 0.90, and normed fit index (NFI) ≥ 0.90. Statistical significance was set at *p* < 0.05.

## 3. Results

### 3.1. Common Method Bias Detection

To assess potential common method variance, this study performed Harman’s single‐factor test using unrotated principal component analysis. The first extracted factor accounted for 42.31% of the total variance, which was below the 50% threshold, indicating that there was no significant common method variance in our study [42].

### 3.2. Demographic Characteristics

The participants had a mean age of 36.52 years (SD = 6.53), with ages ranging from 23 to 59 years. Among them, 96.5% (*n* = 1030) were female, 86.5% (*n* = 923) were married, and 91.7% (*n* = 978) held a bachelor’s degree. The majority of nurses were not specialist nurse (77.8%, *n* = 830). The demographic and professional characteristics of the participants are detailed in Table 1.

**TABLE 1:** Participant demographic information (*n* = 1067).

Variables	*n* (%)
*Gender*	
Male	37 (3.5%)
Female	1030 (96.5%)

*Marital status*	
Single	103 (9.7%)
Married	923 (86.5)
Divorced	41 (3.8%)

*Educational level*	
Junior college and below	84 (7.9%)
Bachelor’s degree	978 (91.7%)
Master’s degree and above	5 (0.5%)

*Age (years)*	
20–29	110 (10.3%)
30–39	643 (60.3%)
40–49	248 (23.2%)
≥ 50	66 (6.2%)

*Professional experience (years)*	
1–5 years	87 (8.2%)
6–10 years	284 (26.6%)
11 years and above	696 (65.2)

*Specialist Nurse*	
Yes	237 (22.2%)
No	830 (77.8%)

*Shift situation*	
3 shift system (8 h daily)	722 (67.7%)
2 shift system (12 h daily)	345 (32.3%)

*Exposure to continuous or frequent changes*	
Yes	277 (26.0%)
No	790 (74.0%)

*Types of organizational changes*	
Reform nursing service models	242 (87.4%)
Update nursing techniques and equipment	211 (76.2%)
Adjust working systems and processes	218 (78.7%)
Upgrade nursing information systems	239 (86.3%)
Reassign job positions	185 (66.8%)

### 3.3. Scores of Nurse Change Fatigue, Organizational Silence, and Work Engagement

This study showed that there is no significant deviation from normality (skewness and kurtosis values varied between −0.775 and 0.243). The mean scores of change fatigue, organizational silence, and work engagement were 25.40 ± 9.27, 49.76 ± 19.37, and 44.70 ± 13.72, respectively. Detailed scores for each dimension are presented in Table 2.

**TABLE 2:** Mean scores and bivariate correlations analysis between variables (*n* = 1067).

Variables	Total score (*M* ± SD)	Average item score (*M* ± SD)	1	2	3	4	5	6	7	8	9	10
**1. Change fatigue**	25.40 ± 9.27	4.23 ± 1.55	1.000									
**2. Organizational silence**	49.76 ± 19.37	2.49 ± 0.97	**0.440** ^∗^	1.000								
3. Acquiescent silence	15.60 ± 6.19	2.60 ± 1.03	0.425^∗^	0.903^∗^	1.000							
4. Defensive silence	14.88 ± 6.95	2.48 ± 1.16	0.395^∗^	0.950^∗^	0.811^∗^	1.000						
5. Prosocial silence	10.91 ± 4.76	2.73 ± 1.19	0.326^∗^	0.855^∗^	0.672^∗^	0.787^∗^	1.000					
6. Indifference silence	8.37 ± 3.99	2.09 ± 1.00	0.397^∗^	0.778^∗^	0.620^∗^	0.671^∗^	0.543^∗^	1.000				
**7. Work engagement**	44.70 ± 13.72	4.97 ± 1.52	**−0.332** ^∗^	**−0.402** ^∗^	−0.362^∗^	−0.401^∗^	−0.255^∗^	−0.386^∗^	1.000			
8. Vigor	14.64 ± 4.64	4.88 ± 1.55	−0.188^∗^	−0.248^∗^	−0.205^∗^	−0.238^∗^	−0.142^∗^	−0.299^∗^	0.644^∗^	1.000		
9. Dedication	14.87 ± 5.40	4.96 ± 1.80	−0.327^∗^	−0.383^∗^	−0.342^∗^	−0.374^∗^	−0.262^∗^	−0.363^∗^	0.832^∗^	0.576^∗^	1.000	
10. Absorption	15.19 ± 4.76	5.06 ± 1.59	−0.158^∗^	−0.212^∗^	−0.194^∗^	−0.217^∗^	−0.101^∗^	−0.228^∗^	0.711^∗^	0.480^∗^	0.522^∗^	1.000

*Note:* Boldface values indicate correlations between the total scores of the scales; nonbold values indicate correlations between the dimension scores of the scales.

^∗^ *p* < 0.001.

### 3.4. Correlation Analysis of Nurse Change Fatigue, Organizational Silence, and Work Engagement

The Pearson’s correlation analysis among nurse change fatigue, organizational silence, and work engagement is detailed in Table 2. A positive correlation was found between change fatigue and organizational silence (*r* = 0.440, *p* < 0.001). Furthermore, significant negative correlations were found between change fatigue and work engagement (*r* = −0.332, *p* < 0.001) and between organizational silence and work engagement (*r* = −0.402, *p* < 0.001).

### 3.5. The Mediating Effect of Organizational Silence Between Change Fatigue and Work Engagement Among Clinical Nurses

#### 3.5.1. Validation Results of the Model

Based on theoretical literature review and correlation analysis, this study constructed a structural equation model with change fatigue as the independent variable, organizational silence as the mediating variable, and work engagement as the dependent variable. The calculated model fit indices were as follows: *χ* ^2^ = 144.35, df = 18, RMSEA = 0.081, SRMR = 0.045, GFI = 0.966, AGFI = 0.933, CFI = 0.978, TLI = 0.966, and NFI = 0.975. Comprehensive evaluation of these indicators revealed mixed model fit results. Specifically, the *χ* ^2^/df value was 8.02, which exceeded the acceptable threshold of 3–5 for absolute model fit. In contrast, the error‐based fit indices indicated favorable model performance: SRMR reached 0.045, reflecting excellent fit, while RMSEA was 0.081, indicating borderline acceptable fit. All CFIs, including CFI, TLI, and NFI, were well above the 0.90 cutoff standard. Meanwhile, GFI and AGFI also met the recommended thresholds, verifying satisfactory explanatory power of the structural model. Overall, despite the suboptimal absolute fit indicated by the elevated *χ* ^2^/df, multiple complementary fit indices collectively confirmed the acceptable overall fit of the constructed model.

#### 3.5.2. Path Analysis

A path coefficient estimation and significance test were performed using SEM to verify the relationships between variables (Table 3). The findings showed that change fatigue positively predicted organizational silence among clinical nurses (*β* = 0.433, *p* < 0.001) and negatively associated with work engagement (*β* = −0.194, *p* < 0.001), thus supporting Hypothesis 1 and Hypothesis 3. Furthermore, organizational silence negatively predicted work engagement (*β* = −0.354, *p* < 0.001), thus supporting Hypothesis 2.

**TABLE 3:** Path analysis.

Path	Effect (unstandardized *β*)	SE	CR	*p*	Effect (standardized *β*)
Change fatigue ⟶ organizational silence	0.245	0.017	14.523	< 0.001	0.433
Change fatigue ⟶ work engagement	−0.289	0.027	−10.532	< 0.001	−0.354
Organizational silence ⟶ work engagement	−0.089	0.015	−6.069	< 0.001	−0.194

Abbreviations: CR = critical ratio, SE = standard error.

#### 3.5.3. Effect Analysis

**FIGURE 1:**
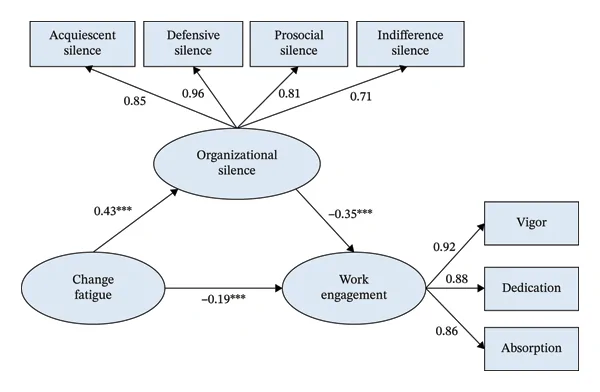
| Structural equation model of change fatigue, organizational silence, and work engagement. ^∗∗∗^ *p* < 0.001.

The bootstrap method with 5000 samples was used to determine the mediating effect of organizational silence in the relationship between the nursing change fatigue and work engagement (Table 4). The findings indicate that change fatigue is directly and significantly related to nurse work engagement (*β* = −0.194, *p* < 0.001). The indirect effect through organizational silence was found to be *β* = −0.153, which was also statistically significant (*p* < 0.001). The 95% CI for the indirect effect was (−0.089, −0.054); since the interval did not include zero, the presence of a mediating effect is reliably supported. A path model with organizational silence as the mediating variable was constructed in this study, as presented in Figure 1. Additionally, the possibility of multicollinearity was assessed using the variance inflation factor (VIF), and all variables were found to have VIF values below 3. This finding indicates that multicollinearity is not a concern in the model (Table 4). The total effect was found to be *β* = −0.347 (*p* < 0.001, 95% CI: −0.189, −0.130). Hence, the indirect effect accounts for 44.1% of the total effect, supporting Hypothesis 4. In summary, organizational silence partially mediated the relationship between change fatigue and work engagement among Chinese clinical nurses.

**TABLE 4:** The mediating effect of organizational silence in the relationship between change fatigue and work engagement among clinical nurses.

Mediation model	*B*	SE	*β*	VIF	*p*	95% CI	Effect proportion (%)
Total effect	−0.160	0.015	−0.347		< 0.001	−0.189, −0.130	—
Direct effect	−0.089	0.015	−0.194	1.28	< 0.001	−0.118, −0.061	55.9
Indirect effect	−0.071	0.008	−0.153	1.28	< 0.001	−0.087, −0.055	44.1

Abbreviations: CI = confidence interval, SE = standard error.

## 4. Discussion

### 4.1. The Current Status of Change Fatigue, Organizational Silence, and Work Engagement Among Chinese Clinical Nurses

#### 4.1.1. Change Fatigue

The total change fatigue score among nurses in this study was 25.40 ± 9.27, with an average item score of 4.23 ± 1.55. This finding indicates that change fatigue is prevalent in the nursing workforce and demands urgent attention. In China, ongoing healthcare system reforms designed to address an aging population and a rising disease burden require nurses to adapt to frequent changes, such as new workflows, technological updates, and modifications to information systems, making them particularly susceptible to change fatigue. This score was lower than that reported by Wang et al. for ICU nurses (26.25 ± 7.06) [43] and higher than that reported by Zhang et al. for 21,588 general nurses (23.62 ± 7.98) [44]. These differences may be attributed to variations in study population, change frequency, change readiness, hospital characteristics, and supportive resources. Unlike change resistance, change fatigue does not necessarily manifest as overt opposition; even when nurses experience high levels of fatigue in the face of change, they may still continue to support the changes. Consequently, nurses’ change fatigue is easily overlooked by nursing managers in the fast‐evolving healthcare environment, they often regard it as a normal part of nursing work [19]. Beaulieu et al. suggest that understanding nurses’ experience of change fatigue is essential, as it has the potential to undermine the direction of organizational change [23]. Therefore, targeted mitigation strategies should be provided to assuage change fatigue, including regularly evaluating nurses’ change fatigue levels, enhancing change readiness, and arranging the pace of change.

#### 4.1.2. Organizational Silence

The overall average score of organizational silence among enrolled nurses was 49.76 ± 19.37, reflecting a moderate level of organizational silence, which aligns with findings from previous cross‐sectional investigations [45, 46]. Organizational silence commonly exists as an adverse long‐term issue in clinical nursing, whereby nurses refrain from voicing workplace worries. Given nursing’s high‐risk and high‐accountability attributes, open communication is essential for safe clinical practice. In this study, the prosocial silence dimension yielded significantly higher item scores (2.73 ± 1.19) relative to other subdimensions. A possible explanation for this result is that nursing is a highly collaborative occupation. To avoid embarrassing colleagues, harming ward harmony and team stability when identifying flawed change arrangements, inefficient workflows, or resource shortages, nurses tend to remain silent out of prosocial motives. Existing literature confirms that organizational silence hinders smooth organizational change [30]. A culture of silence inhibits medical error reporting, impedes internal communication channels, and compromises nursing care quality [47]. Therefore, it is essential for managers to evaluate and curb the prevalence of silent behaviors among nurses so as to sustain institutional operational efficiency amid the fast‐evolving landscape of the healthcare sector.

#### 4.1.3. Work Engagement

The mean total score of nurses’ work engagement was 44.70 ± 13.72, indicating a high level of work engagement among the recruited nursing participants, and this score was higher than the result reported by Jia et al. [11]. Consistent with prior research [6], the present study found that the vigor dimension obtained the lowest score. One plausible cause is that the study sample mainly consisted of nurses from Grade A tertiary hospitals. These medical institutions impose heavy workloads and high work intensity, which continuously exhaust nurses’ physical and mental resources and prevent effective recovery, gradually weakening their persistent vitality at work. Higher work engagement correlates with better job satisfaction, enhanced personal fulfilment, and reduced risk of occupational burnout. Sufficient work engagement enables nurses to explore the intrinsic meaning of their profession, realize personal value, and maintain lasting enthusiasm for clinical work [48]. However, nursing is an inherently high‐stress occupation: frontline nurses regularly adapt to volatile working environments, coordinate multiple concurrent tasks, and bear excessive workload pressures. Such occupational strains tend to deplete nurses’ passion for work, which further highlights the essential role of improving nurses’ work engagement.

### 4.2. The Mediating Role of Organizational Silence in the Relationship Between Change Fatigue and Work Engagement Among Clinical Nurses

This study identified a significant negative association between change fatigue and work engagement among Chinese clinical nurses. Specifically, nurses with higher change fatigue were more likely to exhibit lower work engagement. This result is consistent with the core tenets of the JD‐R model, which proposes that excessive job demands (e.g., additional workload and frequent organizational changes) in conjunction with inadequate job resources (e.g., insufficient organizational and managerial support) may be linked to relatively lower work engagement. Organizational changes require nurses to invest additional time and energy in adapting to updated work requirements [49]. Change fatigue is essentially a negative state caused by excessive depletion of resources. Such a high‐demand and low‐resource working condition continuously depletes nurses’ psychological and professional resources and aggravates negative emotional states, consequently weakening their intrinsic work motivation. As a critical chronic workplace stressor, change fatigue further undermines nurses’ motivational potential, which is reflected in decreased professional enthusiasm, work initiative, and job dedication [50].

Our study revealed that there is a significant negative association between organizational silence and work engagement among nurses, which is consistent with previous research [17]. This result aligns with theoretical perspectives indicating that nurses who experience higher levels of organizational silence tend to report lower work engagement. Organizational silence stems from multiple workplace factors, including hierarchical structures, power imbalances, and insufficient psychological safety [51]. Notably, Chinese nurses are also influenced by collectivist and Confucian cultural values. In a cultural context that emphasizes authority respect and obedience, nurses are reluctant to disclose their psychological distress, which aggravates internal stress and further depletes their psychological resources. When nurses perceive that their voices are devalued or that speaking up cannot bring positive changes, they become less motivated to engage actively in team collaboration and quality improvement efforts for patient care [48]. In addition, voicing personal opinions may potentially impose extra workload and interpersonal pressure on frontline clinical nurses, which could encourage them to adopt organizational silence as a passive stress‐coping strategy [52]. This phenomenon may originate from a prevalent workplace misconception that “the more one speaks up, the more workload one receives; the less one voices opinions, the fewer burdens one undertakes.”

The study further explored the potential mediating role of organizational silence in the linkage between change fatigue and work engagement. The findings suggest that change fatigue may exert both direct and indirect predictive effects on nurses’ work engagement. With change fatigue, nurses become disengaged and apathetic to the change and do not express their dissent even though it is explicitly felt. This result underscores a potential need for nursing managers to attend to fatigue issues arising during organizational change processes. This study verified that organizational silence serves as a partial mediator in the pathway from change fatigue to work engagement. In other words, although change fatigue can directly influence nurses’ work performance and engagement, a considerable proportion of this relationship may function through nurses’ silent behaviors. These findings are consistent with prior research indicating that nurses tend to remain silent in unfavorable practice environments [51]. Furthermore, organizational silence may exacerbate the adverse outcomes associated with change fatigue. Such a workplace climate could discourage nurses from disclosing job burnout or seeking support, which may reduce their work motivation and organizational commitment, and potentially lower their work investment [53]. These outcomes further support the value of exploring organizational silence as a critical underlying mechanism for improving nurses’ work engagement.

### 4.3. Limitations

This study has several limitations to note. First, recruitment through WeChat‐based convenience sampling may have introduced self‐selection bias. The sample may contain a higher proportion of nurses with greater change fatigue and organizational silence, while other nurses may be underrepresented. This may have influenced the average levels reported for change fatigue, organizational silence, and work engagement. It may also have affected the magnitude of the associations observed between variables (for example, if the sample is clustered within similar workplaces). In this study, the moderate positive correlation between change fatigue and organizational silence (*r* = 0.440) might be inflated by this sampling bias. Similarly, the negative correlations of these two constructs with work engagement may also be overestimated. For these reasons, the results should be interpreted with caution and confirmed in future studies using broader, multiregion samples and more systematic recruitment strategies. Second, although associations were observed between change fatigue and work engagement, the cross‐sectional nature of the survey prevents any causal interpretation, limiting the extent to which these findings can be generalized. Therefore, longitudinal tracking studies are required in future research to verify the causal pathways between variables more rigorously. Third, although the CFIs (CFI = 0.978 and TLI = 0.966) and the standardized SRMR = 0.045 indicated acceptable relative fit, the *χ* ^2^/df = 8.02 exceeded conventional thresholds (< 5). This value can be partially explained by the large sample size, which inflates the chi‐square statistic, but sample size alone does not entirely account for the magnitude observed. Minor model misspecifications or unmodeled complexities may also have contributed. We recommend that future studies consider potential model refinement or the testing of alternative factor structures. Despite this, the structural path coefficients were interpreted with caution, and the acceptable CFIs support the relative validity of the model.

### 4.4. Implications for Nursing Practice

This study recruited participants from a single city in northwestern China, a region characterized by underdeveloped economic conditions and inadequate medical resources relative to eastern Chinese regions. Furthermore, the unique cultural and institutional attributes of China’s healthcare system, including hierarchical decision‐making mechanisms and rapid policy‐driven institutional reforms, may shape nurses’ change fatigue and organizational silence in ways that differ from other cross‐cultural contexts. The implications discussed in the following are, therefore, context specific rather than universally applicable, and they should be interpreted with appropriate caution.

Given the institutional realities of the Chinese healthcare system, several possible strategies warrant consideration: (a) nursing managers may find it beneficial to adopt people‐oriented, long‐term change management approaches that minimize the risk of staff overburdening; (b) prechange consultations with frontline nurses can help clarify change goals and reduce contextual uncertainty, while systematic training, professional mentoring, and timely feedback during implementation may further support staff adaptation; (c) regular assessment of change fatigue could assist managers in identifying nurses’ practical difficulties and emotional needs; and (d) additionally, improving the transparency of change‐related information and addressing staff concerns in a timely manner may mitigate feelings of powerlessness and ambivalence that arise from information asymmetry. Regarding the mediating role of organizational silence, (a) establishing nonpunitive reporting mechanisms and standardized feedback channels could encourage nurses to voice difficulties and offer professional suggestions with fewer psychological concerns and (b) enhancing nurses’ psychological empowerment, for example, through appropriate delegation of decision‐making authority and positive recognition of proactive contributions, may also strengthen their occupational autonomy and perceived work value. When implemented with attention to specific institutional contexts and practical conditions, such multidimensional strategies hold potential to improve nurses’ work engagement and occupational well‐being; however, their effects are likely to vary, and they should be validated in settings beyond the one examined here.

## 5. Conclusions

This study found that change fatigue and organizational silence were significantly associated with nurses’ work engagement, whereas organizational silence partially mediated between change fatigue and work engagement. Grounded in the COR theory and the JD‐R model, the present findings help elucidate a plausible underlying mechanism. These insights facilitate nurses’ awareness of the adverse implications of passive silence and motivate them to voice their personal needs and professional suggestions during organizational changes.

## Author Contributions

Xiangwen You: conceptualization, data acquisition, data analysis, data interpretation, and writing–original draft.

Haibo Ma: data acquisition, data analysis, writing–review and editing, and visualization.

Rong Yan: conceptualization, investigation, and funding acquisition.

Miao Yao: conceptualization, investigation, and supervision.

Hongyan Lu: methodology, data interpretation, and writing–review and editing.

Xiangwen You and Haibo Ma are co‐authors.

## Funding

This work was supported by Ningxia Natural Science Foundation (no. 2025AAC030835).

## Disclosure

All authors have read and approved the final version of the manuscript.

## Conflicts of Interest

The authors declare no conflicts of interest.

## Data Availability

The data that support the findings of this study are available from the corresponding author upon reasonable request.
